# Retraction: Supramolecular engineering cascade regulates NIR-II J-aggregates to improve photodynamic therapy

**DOI:** 10.1039/d6sc90067d

**Published:** 2026-03-19

**Authors:** Huizhe Wang, Huijia Liu, Wenqing Li, Shuai Li, Jiaqi Zhang, Jingzhe Zang, Li Liu, Peng Wang

**Affiliations:** a Department of Biomedical Engineering, School of Engineering, China Pharmaceutical University Nanjing 210009 China wangpeng@cpu.edu.cn

## Abstract

Retraction of ‘Supramolecular engineering cascade regulates NIR-II J-aggregates to improve photodynamic therapy’ by Huizhe Wang *et al.*, *Chem. Sci.*, 2024, **15**, 11347–11357, https://doi.org/10.1039/D4SC03020F.

The Royal Society of Chemistry, with the agreement of the named authors, hereby wholly retracts this *Chemical Science* article due to concerns with the interpretation of data that impact the reliability of the work as a whole.

After publication, concerns were raised regarding the collection, and interpretation, of the data and a member of the journal editorial board concurred with the assessment and felt that the conclusions of the work were not sufficiently supported by the experimental results. Following discussions with the editorial office, the corresponding author acknowledged errors in the determination of the compounds.

At this time, and to allow time to complete further experiments and data analysis, the authors have decided to withdraw the paper. The authors apologize for these errors.

Huizhe Wang, Huijia Liu, Wenqing Li, Shuai Li, Jiaqi Zhang, Jingzhe Zang, Li Liu and Peng Wang have agreed to the retraction.

Signed: Huizhe Wang, Huijia Liu, Wenqing Li, Shuai Li, Jiaqi Zhang, Jingzhe Zang, Li Liu and Peng Wang, 9th March 2026.

Retraction endorsed by Rebecca Campbell, Executive Editor, *Chemical Science*.

